# Optimization of urban and rural electric bus scheduling considering mobile battery pack deployment

**DOI:** 10.1371/journal.pone.0339387

**Published:** 2026-01-02

**Authors:** Song Chen, Ande Chang, Yuan Cong

**Affiliations:** 1 School of Forensic Science and Technology, Criminal Investigation Police University of China, Shenyang, China; 2 College of Forensic Sciences, Criminal Investigation Police University of China, Shenyang, China; 3 School of Transportation, Jilin University, Changchun, Jilin, China; Sunway University, MALAYSIA

## Abstract

To address the challenges of insufficient battery power and service interruptions faced by electric buses (EBs) on urban and rural electric bus routes, this study proposes an urban and rural electric bus scheduling method considering mobile battery pack (MBP) deployment. This method involves deploying MBPs at one or more designated stations along the upward direction of the route, enabling EBs requiring en-route charging to pick up an MBP when passing through this station and returning it when passing through the corresponding downward stations on the return travel. A 0–1 mixed-integer programming model is formulated to achieve the joint optimization of the MBP deployment scheme, EB scheduling plan, and charging plan, targeting the minimization of the average daily operating cost of the route (including vehicle usage costs, MBP usage costs, charging costs, and battery degradation costs). Subsequently, the formulated model is addressed by employing a genetic algorithm with an elite retention strategy. Finally, a case study based on an actual urban and rural electric bus route is conducted and a comparison is made with the urban and rural electric bus scheduling method without MBPs. The results demonstrate that the urban and rural electric bus scheduling method considering mobile MBP deployment effectively reduces the average daily operating costs. Specifically, vehicle usage costs, charging costs, and battery degradation costs are reduced by 8.11%, 2.93%, and 21.06%, respectively, leading to an overall cost reduction of 5.20%. These findings confirm the significant potential of MBPs in enhancing the flexibility and economic viability of the urban and rural electric bus route.

## 1. Introduction

### 1.1. Background

Electric buses (EBs) offer significant advantages in terms of zero emissions, lower energy consumption, and ease of operation. With the development of carbon peak and carbon neutrality strategy and the implementation of integrated urban and rural public transit systems, EBs have been widely deployed within both urban and rural transit networks. Unlike urban EB routes, urban and rural EB routes exhibit distinct operational conditions: (i) longer inter-terminal distances and trip times; (ii) sparser and more dispersed stop layouts; (iii) longer headways; and (iv) an asymmetric charging infrastructure, with charging resources concentrated at the urban terminal while the rural terminal is often incomplete or lacks chargers. These features raise per-trip energy consumption and reduce feasible charging opportunities, thereby amplifying EB limitations, namely long charging durations and limited driving range, which in turn elevate the risk of mid-route power shortfalls and service interruptions [1]. Moreover, the typical charging strategy currently employed is to concentrate charging when EBs return to the terminal. This strategy has shortcomings such as long charging durations and strict constraints on the vehicle scheduling plan. This may reduce the operating efficiency of the route and necessitate an increased fleet size to meet timetable requirements, thereby raising the operating costs.

The emergence of portable mobile battery packs (MBPS) offers a novel solution to the aforementioned issues. MBPs can be attached to the rear of EBs during operation, enabling EBs to charge en route whilst running. This effectively reduces charging time, avoids service interruptions, and enhances operation continuity. Based on the widespread distribution of commercial facilities (such as convenience stores and supermarkets) along urban and rural bus routes, this study proposes a novel urban and rural electric bus scheduling method considering MBP deployment. Specifically, bus companies can uniformly charge MBPs during nighttime off-peak electricity periods, then deploy the charged MBPs to selected stores near designated stations along the route with the first trip of the following day. During subsequent operations, EB requiring use of the MBP can pick it up when passing through this station on their upward direction of travel and return it when passing through the corresponding downward station on the return travel. Additionally, the last bus of the day will collect all MBPs from the stations and return them to the departure terminal for centralized charging, preparing for the following operation. This method effectively utilises existing resources without adding new fixed charging infrastructure, significantly enhancing the charging flexibility and reliability of the urban and rural electric bus system.

Therefore, this study focuses on the urban and rural electric bus route, aiming to enhance overall operational efficiency. Aiming to minimize average daily operating costs, we formulate a collaborative optimization model to obtain the MBP deployment scheme, EB scheduling plan, and charging plan. The proposed method holds significant importance for strengthening the operational robustness and charging efficiency of the urban and rural electric bus system, offering new solutions and technical support for the development of green, efficient, and intelligent public transit networks in both urban and rural areas.

### 1.2. Literature review

EBs are a vital carrier for facilitating the electrification of transport. In recent years, related studies have conducted extensive explorations in areas such as infrastructure deployment, route planning, timetable development, vehicle scheduling, and charging scheduling [2–6]. Although substantial literature has developed concerning the application and optimization of EBs in urban environments, research on urban and rural public transport systems remains relatively limited [7–12]. Therefore, this section reviews relevant literature from two perspectives: (i) urban and rural public transit; and (ii) electric bus transit.

(i) urban and rural public transit

To address the challenges faced by public transportation operations under the integration of urban and rural public transit systems, existing studies have conducted preliminary explorations from the perspectives of system design, operational mechanisms, and scheduling strategies. Porru et al. [7] examined the comparative implementation of smart mobility strategies in rural versus urban settings and highlighted three key challenges: establishing standardized and dynamic routing metrics, and streamlining the planning and allocation of mobility-related investments. Schlüter et al. [8] assessed the applicability of demand-responsive transit in integrated rural–urban contexts by employing the agent-based simulation platform MATSim to model residents’ travel behavior. Zeng et al. [9] presented an innovative public transportation paradigm that combines passenger and freight services, aiming to simultaneously address travel needs and charging demands while minimizing overall travel expenses. Qu et al. [10] investigated strategies for improving both the operational efficiency and design of public transport systems connecting urban and rural areas. He et al. [11] designed an integrated framework for rural electric transport that jointly serves passenger travel and e-commerce logistics, aiming to minimize user and operator costs and determine the EB routing and scheduling plan.

(ii) electric bus transit

In recent years, with the accelerating electrification of urban public transportation, the development of electric bus systems has gained increasing attention [13–17]. A considerable number of works have been carried out on the vehicle scheduling and charging scheduling for electric bus systems, aiming to coordinate route assignments with vehicle availability under energy and operational constraints [18–21]. Jahic et al. [22] formulated a mixed-integer programming model for EB scheduling under heterogeneous battery capacities, aiming to determine optimal fleet composition and size. Combining battery swapping and plug-in fast charging, Huang and Wang [23] proposed a hybrid charging strategy to address the uncertainty of battery energy consumption. Considering battery degradation effect and nonlinear EB charging profile, Zhou et al. [24] established a model to collaboratively optimize EB scheduling plan and charging plan under the partial charging policy, while Jin et al. [25] formulated a model to determine the EB charging plan. Bie et al. [26] designed a hybrid energy supply framework combining photovoltaic generation, energy storage, and the power grid to address photovoltaic output volatility and further formulated an EB scheduling optimization model under scenario-specific charging strategies. Incorporating en-route mobile battery swapping into the electric-vehicle routing framework, Xiao et al. [27] formulated an optimization model to minimize the purchase costs of electric vehicles and battery swapping vans and distance-dependent operating costs. Considering fleet attributes, itinerary time windows, charger capacity constraints, dynamic electricity prices, and partial charging, Naeimian et al. [28] formulated an EB en-route charging plan optimization model. Zhang et al. [29] established an EB scheduling plan optimization model to minimize the charger investment costs, EB charging costs, and fleet composition costs, taking into account heterogeneous fleets and chargers, partial charging strategy, and charger-vehicle compatibility.

Some studies have attempted to jointly address multiple planning tasks rather than treating them separately, aiming to improve both the efficiency and the cost-effectiveness of the public transport system [30–33]. Under the opportunity charging strategy, Wang et al. [34] developed a coordinated optimization method to determine the battery rated capacity, EB fleet size, EB scheduling plan, EB charging plan, and charger deployment scheme, aiming to minimize the overall operation costs. McCabe et al. [35] formulated an optimization model to obtain the charger placement, EB charging schedules, and the number of chargers by balancing infrastructure investment and operational efficiency. Aimed at minimizing total operating cost, He et al. [36] presented a comprehensive optimization framework for the EB system, integrating charging infrastructure deployment, EB scheduling, and charging scheduling into a nonlinear mixed-integer programming model. Li et al. [37] designed an integrated framework for optimizing the EB charging plan, battery rated capacity, and dynamic wireless charging facility deployment scheme under dynamic wireless charging mode. Nath et al. [38] investigated the joint optimization of charging station location, vehicle-to-trip assignment, and EB charging time/location decisions, and addressed large-scale problem instances using an iterated local search heuristic. Behnia et al. [39] formulated an integrated EB operation optimization model, incorporating the determination of EB fleet size, EB scheduling, and charging infrastructure deployment to minimize operating costs.

In summary, the optimization of the urban and rural electric bus scheduling plan and charging plan still faces a significant research gap. In particular, existing studies have not fully considered the potential of mobile charging resources (e.g., MBPs) in alleviating charging bottlenecks and enhancing scheduling flexibility. The key challenge lies in achieving optimal coordination between limited charging resources and complex route structures, while ensuring operational continuity. Therefore, this study proposes an urban and rural electric bus scheduling method considering MBP deployment.

### 1.3. Contributions

Three main contributions are made by this research:

(i) We present an urban and rural electric bus scheduling method that incorporates the deployment of MBPs. This method entails the deployment of MBPs at designated stations, enabling EBs to remove these MBPs from the station on their upward direction of travel and attach them to the EB during transit for en-route charging, and to return them upon subsequent passage through the corresponding downward station on the return travel. This method can effectively alleviate scheduling bottlenecks caused by limitations of traditional charging methods, thereby reducing the fleet size required for operations. Concurrently, this method appropriately reduces the depth of discharge (DOD) of the battery, thereby slowing the battery degradation rate.
(ii) We formulate a 0–1 mixed-integer programming model to minimize the average daily operating costs of the route, taking into account time-of-use electricity tariff and battery degradation. This cost comprises vehicle usage costs, MBP usage costs, charging costs, and battery degradation costs. Subsequently, we employ the genetic algorithm based on an elite retention strategy to address the model, yielding the optimal MBP deployment scheme, EB scheduling plan, and charging plan.
(iii) We carry out a case study of an actual urban and rural electric bus route to validate the effectiveness of the proposed urban and rural electric bus scheduling method considering MBP deployment. Then, we compare its performance to the urban and rural electric bus scheduling method without MBPs.

The structure of this study is arranged as follows. In Section 2, the development process and solution algorithm for the urban and rural electric bus scheduling model considering mobile battery deployment are presented. Section 3 provides a case study using a real urban and rural electric bus route. The research conclusions and potential future work are outlined in Section 4.

## 2. Methodology

### 2.1. Problem description

**Fig 1:**
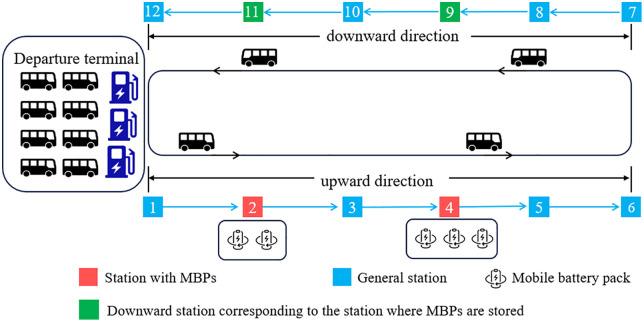
Illustration of the urban and rural electric bus route considering MBP deployment.

This study investigates the bus transit services provided by an urban and rural electric bus route using MBPs during a single day of operation. The service horizon is divided into *T* time slots of 1 minute each, with t(t=1,2,⋯,T) denoting the time index. MBPs are deployed at one or more designated stations along the upward direction of the route (from the urban-side departure terminal toward the rural-side terminal). Fig 1 presents an illustration of the urban and rural electric bus route considering MBP deployment. The urban-side departure terminal (denoted as *o*) is equipped with charging piles, whereas the rural-side terminal has no charging piles. A single bus trip refers to the process where an EB departs from the urban-side departure terminal, travels to the rural-side terminal station, and then returns to the urban-side departure terminal. MBPs are carried by the first EB departing from the urban-side departure terminal and unloaded upon arrival at the designated stations. During subsequent operations, EB requiring use of the MBP can pick it up when passing through these stations on their upward direction of travel and return it when passing through the corresponding downward stations on the return travel. For example, in Fig 1, an EB can pick up an MBP at station 4 on the upward direction of travel and return it at the corresponding downward station (station 9) during the return travel. Additionally, the last bus of the day will collect all MBPs from the stations and return them to the departure terminal for centralized charging, preparing for the following operation.

**Fig 2:**
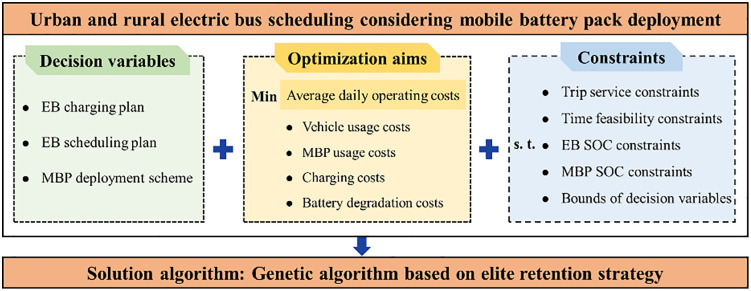
Schematic illustration of the methodological framework.

This study aims to minimize average daily operating costs and determine the optimal MBP deployment scheme, EB scheduling plan, and charging plan based on a given timetable. Fig 2 provides a schematic illustration of the methodological framework. The notations utilized in this section are outlined in Table 1.

**Table 1:** Outline of notation.

Name	Description
**Sets**	
N={1,2,...,N}	Set of stations on the route
I={1,2,...,I}	Set of trips
M={1,2,...,M}	Sets of MBPs equipped on the route
K={1,2,...,K}	Sets of EBs equipped on the route
**Indices**	
n∈N	Index of stations on the route, n∈N
i∈I	Index of trips, i∈I
m∈M	Index of MBPs equipped on the route, m∈M
k∈K	Index of EBs equipped on the route, k∈K
t(t=1,2,⋯,T)	Index of time, where *T* represents the service horizon
q(q=1,2,...,Q)	Index of time period under the time-of-use electricity tariff scheme, where *Q* represents the total number of periods
a(k)(a(k)=1,2,...,A(k))	Index of the charge and discharge cycle for EB *k* within a day, where A(k) denotes the total number of charge and discharge cycles experienced by EB *k* in one day
**Parameters**	
$S_{\max}^{EB}$	Upper limit of battery SOC (%)
$S_{\min}^{EB}$	Lower limit of battery SOC (%)
$S_{j}^{k}$	SOC of EB *k* at the departure time of trip *j* (%)
$S_{\max}^{moblie}$	Upper limit of MBP SOC (%)
$S_{\min}^{mobile}$	Lower limit of MBP SOC (%)
$S_{m}^{end}$	SOC of MBP *m* at the end of daily operation (%)
$S_{initial}$	Battery SOC at the beginning of charging or discharging (%)
$S_{final}$	Battery SOC at the end of charging or discharging (%)
$S_{avg}$	Average SOC during the charge and discharge cycle (%)
$S_{dev}$	Deviation of SOC from the average during the charge and discharge cycle (%)
$S_{a(k)}$	Initial SOC of EB *k* at the beginning of the a(k)−th charge and discharge cycle (%)
${\overset{―}{S}}_{a(k)}$	SOC of EB *k* at the completion of the discharge stage in the a(k)−th cycle (%)
${\overset{―}{\overset{―}{S}}}_{a(k)}$	SOC of EB *k* at the completion of the charging stage in the a(k)−th cycle (%)
$\zeta (S_{initial},S_{final})$	Capacity degradation rate associated with an SOC transition from $S_{initial}$ to $S_{final}$ during a charging or discharging phase (%)
$\xi_{EB}^{k}$	SOH of the battery of EB *k* (%)
$\xi_{mobile}^{m}$	SOH of MBP *m* (%)
$P_{cha}$	Charging power of the charging piles at the departure terminal (kW)
$P_{mob}$	Charging power of MBPs (kW)
$B_{rated}^{k}$	battery rated capacity of EB *k* (kWh)
$B_{rated}^{mobile}$	Rated capacity of MBP *m* (kWh)
$B_{a(k)}$	Degradation capacity of the battery during the a(k)−th cycle (kWh)
$B_{m,\deg}$	Degradation capacity of MBP *m* (kWh)
$c_{battery}$	Purchase costs of the battery (CNY)
$c_{a(k)}$	Degradation cost of the battery during the a(k)−th cycle (CNY)
$c_{eb,pur}$	Average daily procurement cost of an EB (CNY)
$c_{mo,pur}$	Average daily procurement cost of an MBP (CNY)
$c_{q}$	Electricity price in time period *q* (CNY/kWh)
$c_{night}$	Off-peak nighttime electricity price (CNY/kWh)
$t_{j}^{start}$	Departure time of trip *j*
$t_{i}^{end}$	Ending time of trip *i*
$T_{i,ocha}^{k,q}$	Charging duration of EB *k* at departure terminal *o* after completing trip *i* during time period q (min)
$T_{i,mcha}^{k,m}$	Charging duration of EB *k* with MBP *m* during the execution of trip *I* (min)
$T(n,n^{\prime })$	Travel time for an EB to move from station *n* where MBP *m* is stored to the corresponding downward station $n^{\prime }$ (min)
$W_{i}^{o,n^{\prime }}$	Energy consumption of an EB traveling from departure terminal *o* to station $n^{\prime }$ when performing trip *i* (kWh)
**Decision Variables**	
$x_{i,j}^{k}$	If EB *k* consecutively performs trips *i* and *j*, then binary variable $x_{i,j}^{k}=1$; otherwise, $x_{i,j}^{k}=0$
$y_{i}^{k}$	If EB *k* charges at the departure terminal after completing trip *i*, the binary variable $y_{i}^{k}=1$; otherwise, $y_{i}^{k}=0$
$u_{n}^{m}$	If MBP *m* is stored at station *n* during daytime operations, then $u_{n}^{m}=1$; otherwise, $u_{n}^{m}=0$
$d_{i,n}^{k,m}$	If EB *k* requires retrieving MBP *m* stored at station *n* for charging during trip *i*, let 0-1 variable $d_{i,n}^{k,m}=1$; otherwise, $d_{i,n}^{k,m}=0$
**Auxiliary Variables**	
$\tau_{n,g}^{m,k,i}(t)$	If at time *t* the EB *k* uses MBP *m* for charging en route while executing trip *i*, and this is the *g*-th time MBP *m* has been used, let $\tau_{n,g}^{m,k,i}(t)=1$; otherwise, $\tau_{n,g}^{m,k,i}(t)=0$
$S_{n,g}^{m,k}(t)$	SOC of MBP *m* when it is used for the *g*-th time (%)

Let I={1,2,...,I} denote the set of trips, which are indexed as i∈I. Let K={1,2,...,K} denote the sets of EBs equipped on the route, respectively, which are indexed as k∈K. To describe the assignment of trips to EBs, we define a set of binary decision variables. Specifically, if EB *k* consecutively performs trips *i* and *j*, then binary variable $x_{i,j}^{k}=1$; otherwise, $x_{i,j}^{k}=0$, where i,j=1,2,...,I. If trip *j* is the first trip served by EB *k*, then $x_{o,j}^{u}=1$; otherwise, $x_{o,j}^{u}=0$. Similarly, if trip *i* is the last trip performed by EB *k*, then $x_{i,o}^{k}=1$; otherwise, $x_{i,o}^{k}=0$. Furthermore, if EB *k* charges at the departure terminal after completing trip *i*, the binary variable $y_{i}^{k}=1$; otherwise, $y_{i}^{k}=0$.

Let M={1,2,...,M} denote the sets of MBPs equipped on the route, respectively, which are indexed as m∈M. Let N={1,2,...,N} represent the set of stations on the route, which are indexed as n∈N. For the deployment of MBPs, if MBP *m* is stored at station *n* during daytime operations, then $u_{n}^{m}=1$; otherwise, $u_{n}^{m}=0$. Therefore, the total number of MBPs stored at station *n* is $\sum_{m\in M}u_{n}^{m}$. Moreover, if EB *k* requires retrieving MBP *m* stored at station *n* for charging during trip *i*, let 0-1 variable $d_{i,n}^{k,m}=1$; otherwise, $d_{i,n}^{k,m}=0$.

### 2.2. Battery remaining power calculation

#### 2.2.1. EB battery remaining power calculation.

To prolong the service life of batteries, the state of charge (SOC) of EBs during operation is maintained within the interval $\left[S_{\min}^{EB},S_{\max}^{EB}\right]$ [40]. At the start of daily operation, the initial SOC of each EB is set to $S_{\max}^{EB}$. The SOC of EB *k* at the departure time of trip *j*, denoted as $S_{j}^{k}$,can be categorized into five scenarios for discussion:

(i) Trip *j* is the first trip performed by EB *k*, i.e., $x_{o,j}^{k}=1$, $S_{j}^{k}$ can be calculated as:

$$S_{j}^{k}=S_{\max}^{EB},\;x_{o,j}^{k}=1$$ (1)

(ii) EB *k* does not charge en route using the MBP during the execution of trip *i*. After finishing trip *i*, EB *k* also does not charge at the departure terminal and then directly proceeds to execute trip *j*, i.e., $x_{i,j}^{k}=1$, $y_{i}^{k}=0$, and $d_{i,n}^{k,m}=0$. $S_{j}^{k}$ can be computed as:

$$S_{j}^{k}=S_{i}^{k}-W_{i}/B_{rated}^{k}\xi_{EB}^{k},\;x_{i,j}^{k}=1\;and\;y_{i}^{k}=0\;and\;d_{i,n}^{k,m}=0$$ (2)

where $B_{rated}^{k}$ denotes the battery rated capacity of EB *k*, kWh; $\xi_{EB}^{k}$ represents the state of health (SOH) of the battery of EB *k,* %.

(iii) During the execution of trip *i*, EB *k* does not charge en route using the MBP, but instead charges at the departure terminal after finishing trip *i*, and subsequently proceeds to execute trip *j.* In this case, $x_{i,j}^{k}=1$, $y_{i}^{k}=1$, and $d_{i,n}^{k,m}=0$. Accordingly, $S_{j}^{k}$ can be calculated as:

$$S_{j}^{k}=S_{i}^{k}-\frac{W_{i}}{B_{rated}^{k}\xi_{EB}^{k}}+\sum_{q=1}^{Q}\frac{P_{cha}T_{i,ocha}^{k,q}}{60B_{rated}^{k}\xi_{EB}^{k}},\;x_{i,j}^{k}=1\;and\;y_{i}^{k}=1\;and\;d_{i,n}^{k,m}=0$$ (3)

where $P_{cha}$ denotes the charging power of the charging piles at the departure terminal, kW; $T_{i,ocha}^{k,q}$ indicates the charging duration of EB *k* at departure terminal *o* after completing trip *i* during time period q(q=1,2,...,Q), min, where *Q* represents the total number of periods defined under the time-of-use electricity tariff scheme.

(iv) EB *k* charges en route using the MBP during the execution of trip *i.* However, after finishing trip *i*, EB *k* does not charge at the departure terminal and then directly proceeds to execute trip *j*, i.e., $x_{i,j}^{k}=1$, $y_{i}^{k}=0$, and $d_{i,n}^{k,m}=0$. $S_{j}^{k}$ can be calculated as:

$$S_{j}^{k}=S_{i}^{k}-\frac{W_{i}}{B_{rated}^{k}\xi_{EB}^{k}}+\frac{P_{\text{2}}T_{i,mcha}^{k,m}}{60B_{rated}^{k}\xi_{EB}^{k}},\;x_{i,j}^{k}=1\;and\;y_{i}^{k}=0\;and\;d_{i,n}^{k,m}=1$$ (4)

where $T_{i,mcha}^{k,m}$ denotes the charging duration of EB *k* with MBP *m* during the execution of trip *i*, min.

(v) EB *k* charges en route using the MBP during the execution of trip *i*, and also charges at the departure terminal after performing trip *i*, and then proceeds to execute trip *j*, namely, $x_{i,j}^{k}=1$, $y_{i}^{k}=1$, and $d_{i,n}^{k,m}=1$. $S_{j}^{k}$ can be calculated as Eq. (5):

$$S_{j}^{k}=S_{i}^{k}-\frac{W_{i}}{B_{rated}^{k}\xi_{EB}^{k}}+\frac{P_{\text{2}}T_{i,mcha}^{k,m}}{60B_{rated}^{k}\xi_{EB}^{k}}+\sum_{q=1}^{Q}\frac{P_{cha}T_{i,ocha}^{k,q}}{60B_{rated}^{k}\xi_{EB}^{k}},\;x_{i,j}^{k}=1\;and\;y_{i}^{k}=1\;and\;d_{i,n}^{k,m}=1$$ (5)

#### 2.2.2. MBP remaining power calculation.

To describe the dynamic evolution of the MBP SOC, an auxiliary binary variable $\tau_{n,g}^{m,k,i}(t)$ is introduced. Specifically, if at time *t* the EB *k* uses MBP *m* for charging en route while executing trip *i*, and this is the *g*-th time MBP *m* has been used, let $\tau_{n,g}^{m,k,i}(t)=1$; otherwise, $\tau_{n,g}^{m,k,i}(t)=0$. The SOC of MBP *m* when it is used for the *g*-th time, denoted as $S_{n,g}^{m,k}(t)$, is calculated as shown in Eq. (6).


$$S_{n,g}^{m,k}(t)=\left\{ \begin{array}{c} S_{\max}^{moblie},\;g=1\\ S_{n,g-1}^{m,k^{\prime }}(t^{\prime })-\tau_{n,g-1}^{m,k^{\prime },i^{\prime }}(t^{\prime })\frac{P_{2}T_{i^{\prime },mcha}^{k^{\prime },m}}{60B_{rated}^{mobile}\xi_{mobile}^{m}},\;g>1\;and\;\tau_{n,g-1}^{m,k^{\prime },i^{\prime }}(t^{\prime })=1\;and\;d_{i^{\prime },n}^{k^{\prime },m}=1 \end{array}\right.$$ (6)

where $S_{n,g-1}^{m,k^{\prime }}(t^{\prime })$ denotes the SOC of MBP *m* when it is used for the (*g*−1)-th time, %; $S_{\max}^{moblie}$ is the maximum SOC value of the MBP and also the initial SOC value of MBP *m* at the start of daily operation, %; $\xi_{mobile}^{m}$ represents the SOH of MBP *m*, %; $B_{rated}^{mobile}$ is the rated capacity of MBP *m*, kWh.

### 2.3. Battery degradation cost calculation

The costs of battery capacity degradation caused by cycle aging constitute a major component of the total operating cost of the route. A complete charge and discharge cycle is defined as the process between the completion of one charging session and the completion of the next charging session. For each cycle, the capacity degradation rate, $\zeta (S_{initial},S_{final})$, can be estimated using the empirical aging model, as shown in Eqs. (7)–(9) [41,42]. Moreover, because the SOC at the onset of charging does not necessarily coincide with that at the end of discharging, the capacity degradation rates during charging and discharging may differ. Therefore, in a charge and discharge cycle, the capacity loss can be expressed as the sum of two terms: the product of the charging throughput and the capacity degradation rate during charging, and the product of the discharging throughput and the capacity degradation rate during discharging. Let A(k) denote the total number of charge and discharge cycles experienced by EB *k* in one day. The degradation capacity of the battery during the a(k)−th cycle (a(k)=1,2,...,A(k)) can be calculated as Eq. (10).


$$\zeta (S_{initial},S_{final})=\gamma_{1}S_{dev}e^{\gamma_{2}S_{avg}}+\gamma_{3}e^{\gamma_{4}S_{dev}}$$ (7)

$$S_{avg}=(S_{initial}+S_{final})/2$$ (8)

$$S_{dev}=(\left|S_{final}-S_{initial}\right|)/2$$ (9)

$$B_{a(k)}=\zeta (S_{a(k)},{\overset{―}{S}}_{a(k)})\times B_{rated}^{k}\xi_{eb}^{k}(S_{a(k)}-{\overset{―}{S}}_{a(k)})+\zeta ({\overset{―}{S}}_{a(k)},{\overset{―}{\overset{―}{S}}}_{a(k)})\times B_{rated}^{k}\xi_{eb}^{k}({\overset{―}{\overset{―}{S}}}_{a(k)}-{\overset{―}{S}}_{a(k)})$$ (10)

where $\gamma_{1}$, $\gamma_{2}$, $\gamma_{3}$, and $\gamma_{4}$ are model parameters; $S_{initial}$ denotes the battery SOC at the beginning of charging or discharging, %; $S_{final}$ is the SOC at the end of charging or discharging, %; $S_{avg}$ denotes the average SOC during the charge and discharge cycle, %; $S_{dev}$ represents the deviation of SOC from the average during the charge and discharge cycle, %; $S_{a(k)}$ represents the initial SOC of EB *k* at the beginning of the a(k)−th charge and discharge cycle, %; ${\overset{―}{S}}_{a(k)}$ and ${\overset{―}{\overset{―}{S}}}_{a(k)}$ denote the SOC of EB *k* at the completion of the discharge stage (i.e., the start of the charging stage) and at the completion of the charging stage in the a(k)−th cycle, respectively, %.

The battery is considered to reach its retirement threshold once its SOH declines to 80% [40]. In other words, the battery is no longer suitable for continued route operation when cumulative degradation capacity reaches 20% of the rated capacity. Therefore, the degradation cost of the battery during the a(k)−th cycle (a(k)=1,2,...,A(k)) can be estimated as Eq. (11):


$$c_{a(k)}=\frac{B_{a(k)}c_{battery}}{B_{rated}^{k}\times 20\%}$$ (11)

where $c_{battery}$ is the purchase costs of the battery, CNY.

### 2.4. Model formulation

This study aims to minimize the average daily operating costs of the route, which includes vehicle usage costs, MBP usage costs, charging costs, and battery degradation costs. On this basis, an urban and rural electric bus scheduling optimization model considering MBP deployment is established to determine the optimal MBP deployment scheme, EB scheduling plan, and charging plan. These objectives are expressed in Eqs. (12) to (16) and are subject to Eqs. (17) to (26).


$$\min Z=Z_{1}+Z_{2}+Z_{3}+Z_{4}$$ (12)

$$Z_{1}=\sum_{k=1}^{K}\sum_{j=1}^{I}c_{eb,pur}x_{o,j}^{k}$$ (13)

$$Z_{2}=\sum_{m=1}^{M}c_{mo,pur}$$ (14)

$$Z_{3}=\sum_{q=1}^{Q}\sum_{k=1}^{K}\sum_{i=1}^{I}c_{q}P_{1}\times (T_{i,ocha}^{k,q}/60)+(\sum_{k=1}^{K}(S_{\max}^{EB}-S_{k}^{end})B_{rated}^{k}\xi_{EB}^{k}+\sum_{m=1}^{M}(S_{\max}^{moblie}-S_{m}^{end})B_{rated}^{mobile}\xi_{mobile}^{m})c_{night}$$ (15)

$$Z_{4}=\sum_{k=1}^{K}\sum_{a(k)=1}^{a(k)}c_{a(k)}$$ (16)

$$s.t.\;\sum_{k=1}^{K}\sum_{i=1}^{I}x_{i,j}^{k}=1,\;\forall j$$ (17)

$$\sum_{k=1}^{K}\sum_{j=1}^{I}x_{i,j}^{k}=1,\;\forall i$$ (18)

$$\sum_{k=1}^{K}\sum_{j=1}^{I}x_{0,j}^{k}=\sum_{k=1}^{K}\sum_{i=1}^{I}x_{i,0}^{k}$$ (19)

$$x_{i,j}^{k}\left(S_{i}^{k}-\frac{W_{i}}{B_{rated}^{k}\xi_{EB}^{k}}-S_{\min}^{EB}\right)\geq 0$$ (20)

$$S_{m}^{end}-S_{\min}^{mobile}\geq 0$$ (21)

$$t_{j}^{start}-t_{i}^{end}-y_{i}^{k}\sum_{q=1}^{Q}T_{i,ocha}^{k,q}\geq 0,\;ifx_{i,j}^{k}=1$$ (22)

$$T_{i,mcha}^{k,m}\leq \min\left(T(n,n^{\prime }),60(S_{\max}^{EB}-(S_{i}^{k}-\frac{W_{i}^{o,n^{\prime }}}{B_{rated}^{k}\xi_{EB}^{k}}))\frac{B_{rated}^{k}\xi_{EB}^{k}}{P_{mob}}\right)$$ (23)

$$L(n,n^{\prime })-L_{\min}\geq 0\;ifu_{n}^{m}=1\;and\;u_{n^{\prime }}^{m}=1,\forall m$$ (24)

$$x_{i,j}^{k},y_{i}^{k},u_{n}^{m},d_{i,n}^{k,m},\tau_{n,g}^{m,k,i}(t)\in \{0,1\}\;\;\forall i,j,k,m,n,t$$ (25)

$$T_{i,mcha}^{k,m},M\in {\mathbb{N}}^{*}$$ (26)

where $c_{eb,pur}$ denotes the average daily procurement cost of an EB, CNY; $c_{mo,pur}$ represents the average daily procurement cost of an MBP, CNY; $c_{q}$ is the electricity price in time period *q*, CNY/kWh; $c_{night}$ refers to the off-peak nighttime electricity price, CNY/kWh; $t_{j}^{start}$ is the departure time of trip *j*; $t_{i}^{end}$ is the ending time of trip *i*; $P_{mob}$ indicates the charging power of MBPs, kW; $T(n,n^{\prime })$ indicates the travel time for an EB to move from station *n* where MBP *m* is stored to the corresponding downward station $n^{\prime }$, min; $W_{i}^{o,n^{\prime }}$ is the energy consumption of an EB traveling from departure terminal *o* to station $n^{\prime }$ when performing trip *i*, kWh; $L(n,n^{\prime })$ is the distance between station *n* and station $n^{\prime }$, km; $L_{\min}$ is the minimum allowable distance between any two stations where MBPs are deployed, km.

Eqs. (13)–(16) represent the calculation methods for the average daily vehicle usage costs, the average daily procurement costs of MBPs, charging costs, and battery degradation costs, respectively. Among them, the average daily procurement costs of MBPs are determined by their total procurement cost and the number of available service days. The MBPs utilized in this paper employ retired power batteries, with an initial SOH of 80% at deployment. When the SOH declines to 60%, the MBPs are considered no longer viable for operation [43]. The MBPs are charged only at night at the departure terminal, while discharge occurs exclusively during daytime operations. Consequently, each MBP experiences one complete charge and discharge cycle every day. Based on this assumption, the degradation capacity of MBP *m* can be calculated using Eq. (27). Once the accumulated degradation capacity reaches $0.2B_{rated}^{mobile}$, the MBP can no longer be used, thereby determining its available service lifetime.


$$B_{m,\deg}=2B_{rated}^{mobile}\xi_{mobile}^{m}(S_{\max}^{moblie}-S_{m}^{end})\zeta (S_{\max}^{moblie},S_{m}^{end})$$ (27)

where $S_{m}^{end}$ denotes the SOC of MBP *m* at the end of daily operation, %.

Eqs. (17) and (18) stipulate that each trip is uniquely served by a single EB. Eq. (19) requires that every EB dispatched from the departure terminal must return after completing its assigned trips. Eq. (20) guarantees that the SOC of EB *k* remains within the interval $\left[S_{\min}^{EB},S_{\max}^{EB}\right]$ during the execution of trip *i*. Eq. (21) ensures that the SOC of MBP *m* remains within the interval $\left[S_{\min}^{mobile},S_{\max}^{mobile}\right]$. Eq. (22) specifies the time feasibility constraint for EB *k* to proceed to perform trip *j* after completing trip *i*. Eq. (23) is the charging duration constraint for EB *k* to charge en route using MBP *m* during the execution of trip *i*. Eq. (24) stipulates that the distance between two stations deployed with MBPs should not be less than the minimum allowable distance. Eqs. (25) and (26) define the bounds of the decision variables in the optimization model.

### 2.5. Solution algorithm

In Section 2.4, the developed model is expressed as a mixed-integer programming problem. Such problems are typically high-dimensional and involve complex constraints, rendering traditional exact algorithms computationally expensive and inefficient for large-scale practical applications. The genetic algorithm, as a heuristic method based on population search, exhibits strong global search capability, which makes it particularly suitable for addressing combinatorial optimization and nonlinear problems. Moreover, the incorporation of an elite retention strategy preserves the best individuals across generations, thereby enhancing convergence stability and reducing the risk of losing high-quality solutions. Therefore, this study employs a genetic algorithm based on an elite retention strategy to tackle the formulated model.

**Fig 3:**
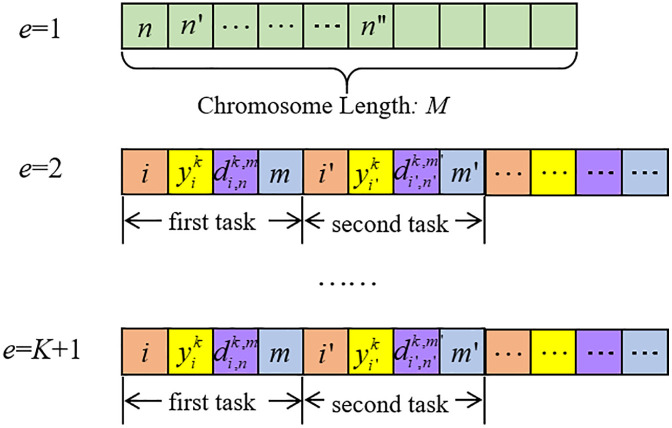
Schematic diagram of chromosome gene coding.

A solution can be obtained through the following steps:

Step 1: Initialize the generation index *g* = 0 and set the maximum number of generations to *G*. The initial population size is defined as *H*, with each feasible individual indexed by h(h=1,2,...,H). Using an integer-encoding scheme, each individual is represented by K+1 chromosomes, indexed by e(e=1,2,...,K,K+1). The first chromosome encodes both the number and locations of deployed MBPs. Its length is equal to the total number of MBPs on the route, i.e., *M*. Each gene position represents one MBP*m*, and the value stored in that gene is the index of its deployment station *n*. In this way, the chromosome specifies the deployment scheme of all MBPs. The remaining *K* chromosomes each correspond to one EB, describing its daily sequence of trips and charging decisions. As illustrated in Fig 3, a chromosome consists of multiple operating tasks, with variable length. Each task is encoded by four basic genes: (i) the trip index performed by the EB; (ii) whether the EB charges at the departure terminal after completing the trip; (iii) whether the EB uses an MBP for charging during the trip; and (iv) the index of the MBP being utilized.

Step 2: Randomly generate the initial population. The number of individuals should be equal to the specified population size *H*. For each chromosome, all genes are required to satisfy the constraints (17) – (26).

Step 3: Define the reciprocal of the objective function as the fitness function. Using Eq. (28), calculate the fitness of each individual in the population sequentially.


$$F_{h}=\frac{\text{1}}{Z_{h}}$$ (28)

Step 4: Check whether the generation index *g* exceeds *G*. If true, move to Step 9; otherwise, continue to Step 5.

Step 5: Selection. A hybrid selection operator combining the elite retention strategy with roulette wheel selection is applied.

Step 5.1: Calculate the fitness of each individual and compute the total fitness of the population.

Step 5.2: Identify the individual with the highest fitness in the current population and record it as the best individual to date.

Step 5.3: Calculate the selection probability of each individual. The probability $P_{h}$ for individual *h* can be calculated as Eq. (29):


$$P_{h}=F_{h}/\sum_{r=1}^{H}F(r)$$ (29)

Step 5.4: Calculate the cumulative selection probability of each individual. To be specific, Eq. (30) shows the calculation method for the cumulative probability $\chi_{h}$ of individual *h*.


$$\chi_{h}=\sum_{r=1}^{H}P_{r}$$ (30)

Step 5.5: Set δ=1. Let $\varsigma_{h}$ denote the number of times individual *h* is selected. Initialize the number of selections for all individuals to 0.

Step 5.6: Produce a random number τ evenly dispersed within the interval [0, 1].

Step 5.7: If $\tau <\chi_{1}$, select individual 1 and update $\varsigma_{1}=\varsigma_{1}+1$. Otherwise, select individual $h^{\prime }$ such that $\chi_{h^{\prime }-1}<\tau \leq \chi_{h^{\prime }}$, and update $\varsigma_{h^{\prime }}=\varsigma_{h^{\prime }}+1$.

Step 5.8: Set δ=δ+1.

Step 5.9: Check whether δ>H. If true, move to Step 5.10; otherwise, return to Step 5.6.

Step 5.10: Rank all individuals in descending order according to the number of times they are selected, and select the top *H* individuals to form the next generation.

Step 5.11: Apply the elite retention strategy, replacing the worst individual in the new generation with the best individual identified to date.

Step 6: Perform crossover operations on individuals in the population with probability $P_{c}$.

Step 6.1: Randomly pair the *H* individuals in the population to form *H*/2 parent pairs. Set ε=1.

Step 6.2: Check whether ε>H/2. If true, move to Step 7; otherwise, continue to Step 6.3.

Step 6.3: Produce a random number evenly dispersed within the interval [0, 1]. Check whether it is less than $P_{c}$, if true, proceed to Step 6.4; otherwise, proceed to Step 6.10.

Step 6.4: Randomly select a gene position from a chromosome of each parent as the crossover point. This position and all subsequent genes are designated as crossover genes. If the selected chromosome is the first chromosome (i.e., representing the MBP deployment scheme), then both parents must use their first chromosomes for crossover, go to Step 6.5; otherwise, proceed to Step 6.6.

Step 6.5: Apply single-point crossover to the first chromosomes of the parents. The resulting MBP deployment scheme may render the associated EB scheduling chromosomes infeasible with respect to operational constraints. Therefore, the EB scheduling plan of the offspring is repaired using the following procedure.

Step 6.5.1: Under the updated MBP deployment scheme, evaluate all trips with respect to SOC constraints and MBP utilization. Any trip that violates SOC bounds, time-window constraints, or MBP energy capacity limits is removed from its original chromosome and added to a repair list.

Step 6.5.2: Attempt to reassign the trips in the repair list to the existing chromosomes (representing the EB scheduling plan) by inserting each trip into feasible positions along the chromosomes. For each tentative insertion, the SOC and MBP energy consumption are updated, and the insertion is accepted only if all constraints remain satisfied.

Step 6.5.3: If a trip cannot be feasibly inserted into any existing chromosomes and the fleet size has not yet reached its upper bound, a new chromosome is created to serve this trip, thereby increasing the fleet size.

Step 6.5.4: Chromosomes with no assigned trips after the repair process are removed from the solution.

Step 6.5.5: If no feasible EB scheduling plan can be obtained under the maximum allowable fleet size, the offspring individual is discarded and the corresponding parent individuals are retained.

Step 6.5.6: Go to Step 6.7.

Step 6.6: Perform crossover on the chromosomes of both parents that represent the EB scheduling plan. Taking as an example the offspring individual that is constructed by inserting the crossover genes from the father into the mother, we describe the crossover operation and the subsequent repair procedure as follows.

Step 6.6.1: Duplicate the mother individual to create a new offspring individual, and remove its crossover genes. Check whether each deleted trip also exists among the crossover genes of the father individual. If so, delete it; otherwise, store the corresponding gene in a temporary list for later reinsertion in Step 6.6.4 so as to maintain the integrity of the offspring individual and guarantee that each trip is executed by a single EB.

Step 6.6.2: Examine the offspring individual to check whether it contains any of the crossover genes of the father individual. If yes, delete the corresponding gene in the offspring individual.

Step 6.6.3: Insert the crossover genes from the father individual into the offspring individual. Verify whether the offspring individual satisfies the constraints. Store any trip indexes that fail to meet the constraints for reinsertion in Step 6.6.4, thereby ensuring the integrity of the newly generated offspring individual.

Step 6.6.4: Search feasible insertion positions for all genes in the temporary list across all chromosomes of the offspring individual, and reinsert a gene only when all constraints are satisfied. Repeat this procedure until no further feasible insertion position can be found for any remaining gene.

Step 6.6.5: If a trip cannot be feasibly inserted into any existing chromosomes and the fleet size has not yet reached its upper bound, a new chromosome is created to serve this trip, thereby increasing the fleet size.

Step 6.6.6: Chromosomes with no assigned trips after the repair process are removed from the solution.

Step 6.6.7: If no feasible EB scheduling plan can be obtained under the maximum allowable fleet size, the offspring individual is discarded, and the corresponding parent individuals are retained.

Step 6.6.8: Go to Step 6.7.

Step 6.7: Let ε=ε+1, proceed to Step 6.2.

Step 7: Apply the mutation to each individual with probability $P_{m}$. Produce a random number τ evenly dispersed within the interval [0, 1]. If the random number is lower than $P_{m}$, randomly select a chromosome from the current individual and modify one of its genes to produce a new individual; otherwise, proceed to the next individual for mutation.

Step 8: Let *g*=*g*+1, move to Step 4.

Step 9: Output the optimal MBP deployment scheme, EB scheduling plan, and charging plan corresponding to the individual with the maximum fitness value.

## 3. Case study

### 3.1. Data investigation

**Fig 4:**
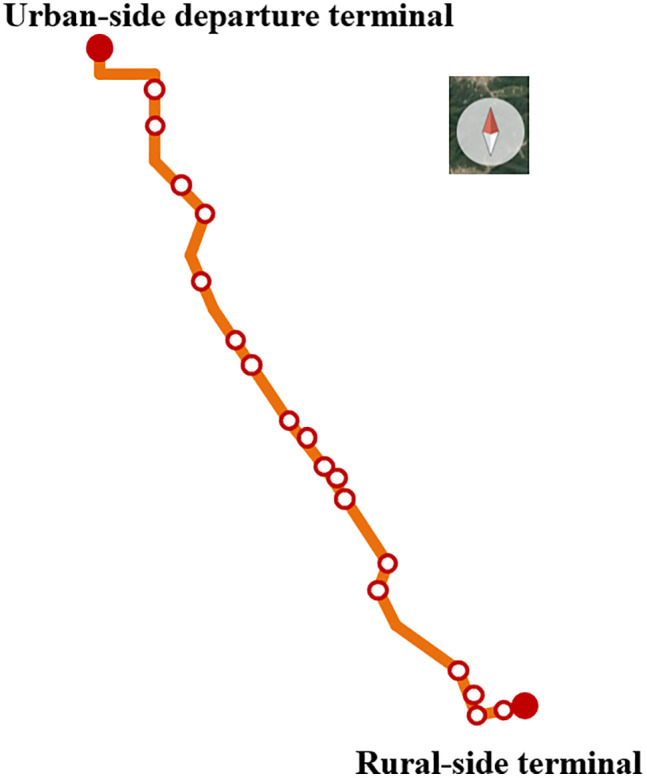
Layout of the urban and rural electric bus route.

To validate the proposed urban and rural electric bus scheduling method considering MBP deployment, this paper adopts a real urban and rural electric bus route in a city in China as a case study. The route spans 58.6 km from terminal *o* to destination, covering 20 stations, with the alignment as illustrated in Fig 4. At the departure terminal, 5 charging piles are installed. As described in Section 2.1, one complete trip consists of an EB traveling from departure terminal *o* to destination and then returning to departure terminal *o*, with a total mileage of 117.2 km. The first bus departs at 5:00, while the last trip begins at 19:00. The operating period is divided into multiple time intervals, with departure headways and travel times summarized in Table 2. Meanwhile, the time-of-use electricity tariff adopted for the case study is reported in Table 3.

**Table 2:** Operational information for each period.

Time period	Dispatching interval (min)	Number of trips	Trip travel time (min)
5:00-7:00	10	12	190
7:00-9:00	5	24	210
9:00-12:00	10	18	190
12:00-15:00	15	12	160
15:00-16:00	10	6	190
16:00-17:00	5	12	210
17:00-18:00	10	6	190
18:00-19:00	15	5	160

**Table 3:** Time-of-use tariff schedule.

Time period	Tariff (CNY/kWh)	Time period	Tariff (CNY/kWh)
6:00-9:00	1.0866	15:30-21:00	1.3574
9:00-11:30	1.3574	21:00-23:00	1.0866
11:30-15:30	1.0866	23:00-6:00	0.8158

A total of 49 EBs are allocated to this route. The vehicle specifications include a curb weight of 11100 kg, external dimensions of 10690 × 2500 × 3200 (mm³), and a rated passenger capacity of 72. Each EB is equipped with a 226 kWh LiFePO_4_ battery, with an average energy density of 142.89 Wh/kg. Based on historical charging data, the SOH of the EB batteries is estimated to be 95%. The average energy consumption rate of an EB without MBPs is 0.914 kWh/km. It is noted that loading one MBP is estimated to increase the energy consumption per kilometer of the EB by approximately 0.058 kWh/km [40]. The inter-station distances along the route are listed in Table 4. Additional model input parameters are summarized in Table 5 [1,43,44].

**Table 4:** Distance between stations along the route.

Station No.	Distance (km)	Station No.	Distance (km)
[1, 2]	4.6	[11, 12]	0.95
[2, 3]	1.95	[12, 13]	1.2
[3, 4]	5.9	[13, 14]	4.93
[4, 5]	2.8	[14, 15]	1.48
[5, 6]	4.85	[15, 16]	11.3
[6, 7]	4.9	[16, 17]	0.8
[7, 8]	1.86	[17, 18]	1.2
[8, 9]	3.8	[18, 19]	1.36
[9, 10]	0.9	[19, 20]	0.86
[10, 11]	2.96		

**Table 5:** Values of some parameter of the model.

Parameter	Value	Parameter	Value
$c_{battery}$	316400 CNY	$S_{\max}^{EB}$	90%
$c_{mo,pur}$	110.60 CNY	$\xi_{mobile}^{m}$	80%
$c_{eb,pur}$	342.47 CNY	$B_{rated}^{mobile}$	300 kWh
$P_{cha}$	120 kW	$S_{\max}^{moblie}$	90%
$P_{mob}$	50 kW	$S_{\min}^{moblie}$	20%
$S_{\min}^{EB}$	10%	$L_{\min}$	5 km

### 3.2. Optimization results

**Fig 5:**
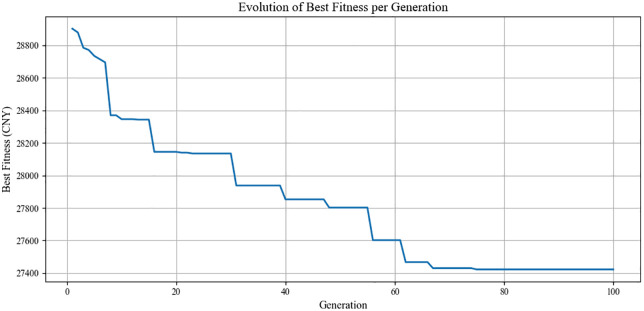
Iteration results.

We solved the proposed model using Python 3.12.5. The numerical experiments were performed on a personal computer equipped with an Intel(R) Core (TM) i9-14900HX CPU @ 2.20 GHz and 32 GB of RAM. In the genetic algorithm, the population size, the maximum number of iterations, the crossover probability, and the mutation probability are set to 100, 100, 0.7, and 0.1, respectively. Fig 5 displays the iteration results. The optimal solution was found within 496 s, which yields the average daily operating costs of 27422.26 CNY, consisting of 11643.98 CNY for vehicle usage costs, 995.61 CNY for MBP usage costs, 10439.09 CNY for charging costs, and 4343.58 CNY for battery degradation costs.

In the optimal solution, a total of 9 MBPs are deployed along the route, with 3 MBPs located at station 7 and 6 MBPs at station 11. Stations 7 and 11 are located approximately 26.86 km and 35.47 km from the urban departure terminal, respectively. After picking up the MBP at these stations, the EB can travel about 63.48 km and 46.26 km with the MBP before reaching the corresponding stations in the downward direction. Based on the average operating speed of EBs, EBs can charge up to 86 kWh and 69 kWh within this period, enough to fulfil the en route charging requirements of most EBs. According to the parameters specified in Section 3.1, the actual usable capacity of each MBP is approximately 168 kWh (300 × 0.8 × 0.7). Consequently, each MBP deployed at station 7 can be used twice by EBs, whereas an MBP located at station 11 can support three uses.

**Fig 6:**
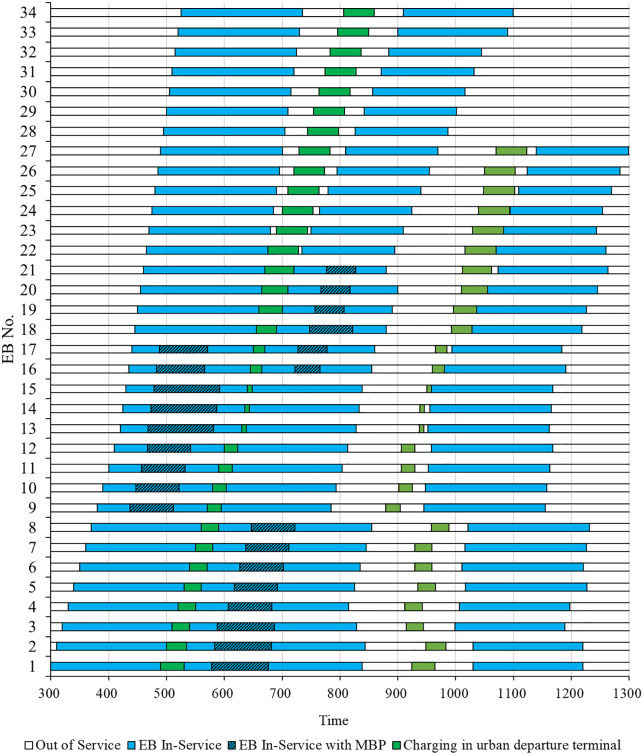
EB scheduling plan and charging plan.

In the optimized operation plan, the number of EBs used is 34, and the EB scheduling plan and charging plan are presented in Fig 6. It should be noted that the figure only reports the charging periods of EB during daytime operations, whereas the final overnight charging process after the last trip is not included in this analysis. In Fig 6, the horizontal axis starts from time 300 (i.e., 05:00), corresponding to the departure of the first scheduled trip. It extends to time 1300 (namely 21:40), which marks the completion of the last trip of the day.

According to Fig 6, EBs typically charge at the departure terminal during the idle period after performing scheduled trips. This is because the route length is relatively long, and the SOC of the EB usually decreases by approximately 50% after completing a trip. Without timely charging, subsequent trips may suffer from insufficient energy, thereby disrupting operational continuity. To address this, MBPs are primarily employed as rapid energy replenishment solutions for EBs undertaking high-frequency trips. In particular, some EBs have short idle intervals between two consecutive trips, during which relying solely on departure terminal charging may be insufficient to restore the SOC to a safe level. As shown in Fig 6, among the EBs that use MBPs for the first time, EB 13, EB 14, and EB 15 exhibit markedly longer MBP-charging durations than the others. This is because the idle time at the urban departure terminal between their first and second trips is extremely short, leaving only a limited charging window and preventing the SOC from being restored to a level that can safely support the subsequent trip. Therefore, during their first trip, EB 13, EB 14, and EB 15 perform en route charging using the MBPs deployed at station 7, which allows for a relatively larger charging capacity.

From the above, the deployment of MBPs can effectively alleviate scheduling bottlenecks arising from the limitations of conventional charging methods, thereby reducing the fleet size. For example, Table 6 illustrates the charging plan of EB 13 involving MBPs. During the execution of trip 13, the EB completes a charging operation via the MBP, replenishing approximately 86 kWh of energy. This maintains the SOC of EB 13 at a high level (78.57%) upon trip completion. Subsequently, EB 13 utilized a 10-minute interval between two successive tasks to perform a short-duration charging at the departure terminal. In contrast, without the support of MBPs, EB 13 can only charge at the depot after completing trip 13. At that time, the SOC drops to 40.11%, and even after 10 minutes of departure terminal charging, it can only increase to 49.43%, which would be insufficient to cover the energy demand of trip 47. This situation would require additional EBs to perform trip 47, thereby increasing overall operating costs.

**Table 6:** Charging plan of EB 13 involving MBPs.

Trip No.	Charging period	Charging mode	Battery SOC before charging	Battery SOC after charging
In trip 13	[7:48, 9:46]	With MBPs	78.57%	90%
After trip 13	[10:30, 10:38]	At the terminal	82.52%	90%
After trip 47	[15:39, 16:33]	At the terminal	40.11%	90.00%
After trip 81	Night charging	At the terminal	40.11%	90.00%

### 3.3. Plan comparison

A comparison is carried out between the proposed urban and rural electric bus scheduling method utilizing MBPs and a baseline schedule without such MBPs, to evaluate its effectiveness. The urban and rural electric bus route operation plan utilizing MBPs is denoted as Plan A, while the urban and rural electric bus route operation plan not employing MBPs is referred to as Plan B. Plan B is generated by solving the same optimization model under the identical algorithmic settings as Plan A, with the number of MBPs fixed at **M* *= 0 (i.e., no MBPs). The comparison results are presented in Table 7. As can be observed through Table 7, the average daily operating cost of Plan A is 1505.55 CNY lower than that of Plan B, representing a reduction of 5.20%. Specifically, compared with Plan B, Plan A achieves reductions of 8.11% in vehicle usage costs, 2.93% in charging costs, and 21.06% in battery degradation costs.

**Table 7:** Comparisons of results.

	Plan A	Plan B	Reduced value (CNY)	Reduced percentage
Total operating costs (CNY)	27422.26	28927.81	1505.55	5.20%
Vehicle usage costs (CNY)	11643.98	12671.39	1027.41	8.11%
Mobile battery pack usage costs (CNY)	995.61			
Charging costs (CNY)	10439.09	10753.73	314.64	2.93%
Battery degradation costs (CNY)	4343.58	5502.69	1159.11	21.06%

In terms of vehicle utilization, compared to Plan B, Plan A requires 3 fewer vehicles in operation, decreasing vehicle operating costs by 1027.41 CNY, with a reduction rate of 8.11%. This benefit mainly arises from the flexibility of the MBPs utilized. MBPs can be integrated into the execution of trips, enabling EBs to conduct en-route charging, thereby preventing service interruptions or vehicle substitutions caused by insufficient SOC. For instance, in the charging plan of EB 13 presented in Table 6, continuous operation across multiple trips is achieved by combining en-route charging and short-interval charging at the departure terminal, thereby eliminating the need for additional reserve EBs. In contrast, under Plan B, EBs rely on charging at the departure terminal during trip intervals. However, when the SOC increases only marginally (e.g., from 40.11% to 49.43%), it cannot meet the energy requirements for the subsequent trip, necessitating the introduction of additional EBs. This contributes to an increase in both the required number of EBs and operating costs.

The charging cost under Plan A shows a slight decrease compared to Plan B, with a reduction of 314.64 CNY, representing a 2.93% decline. This is primarily attributed to the flexibility in charging time management provided by MBPs, which enables the system to transfer some charging demand from peak tariff periods to shoulder hours, thereby reducing charging costs. Such an optimization of the charging time enables Plan A to achieve superior cost performance, even though the total daily energy demand remains essentially unchanged.

In contrast, Plan A demonstrates a more significant reduction in battery degradation costs, amounting to 21.06%. This is because the introduction of MBPs reduces the DOD of the EB battery, thereby effectively slowing the degradation of the battery. However, under Plan B, EB batteries frequently suffer deep DOD cycles or even operate at near-critical SOC levels, thereby accelerating the battery degradation rate. Although Plan A includes an additional CNY 995.61 in MBP usage costs, this expenditure remains significantly lower than the cumulative reduced costs from the aforementioned three items. In short, the application of MBPs substantially enhances the charging flexibility and resource utilization efficiency, demonstrating superior economic adaptability and feasibility.

## 4. Conclusions

This study proposes an urban and rural electric bus scheduling method considering MBP deployment. To minimize the average daily operating costs, a 0–1 mixed-integer programming model is formulated, incorporating coordinated optimization of the MBP deployment scheme, EB scheduling plan, and charging plan. Subsequently, a genetic algorithm based on an elite retention strategy is employed to address the model. Finally, numerical experiments are conducted using an actual urban and rural electric bus route. A comparison is made between the proposed method and the urban and rural electric bus scheduling method without MBPs. The results demonstrate that the deployment of MBPs can avoid service interruptions or vehicle substitutions caused by insufficient SOC, thereby effectively alleviating scheduling feasibility issues arising from limitations in conventional charging methods. Moreover, this strategy can appropriately moderate the DOD of battery charge and discharge cycles, thereby slowing the battery degradation rate. To sum up, the proposed method offers significant advantages in reducing the operating costs of the urban and rural electric bus routes. Specifically, compared with the baseline case without MBPs, vehicle usage costs, charging costs, and battery degradation costs decreased by 8.11%, 2.93%, and 21.06%, respectively, resulting in an overall reduction of 5.20% in average daily operating costs.

Future research could further explore integrating renewable energy into the charging process of MBPs. Additionally, incorporating real-time passenger flow fluctuations and traffic uncertainties into the model could enhance its dynamic adaptability and practical applicability.

## Supporting information

S1 File S1 Table. Operational information for each period. S2 Table. Time-of-use tariff schedule. S3 Table. Distance between stations along the route.
(RAR)
